# First-Principles Study on III-Nitride Polymorphs: AlN/GaN/InN in the *Pmn*2_1_ Phase

**DOI:** 10.3390/ma13143212

**Published:** 2020-07-19

**Authors:** Zheren Zhang, Changchun Chai, Wei Zhang, Yanxing Song, Linchun Kong, Yintang Yang

**Affiliations:** School of Microelectronics, Xidian University, Xi’an 710071, China; zheren950323@gmail.com (Z.Z.); ccchai@mail.xidian.edu.cn (C.C.); syx739686768@163.com (Y.S.); 15129180614@163.com (L.K.); ytyang@xidian.edu.cn (Y.Y.)

**Keywords:** III-Nitride, *Pmn*2_1_ phase, density functional theory, electronic properties, mechanical properties, anisotropic properties

## Abstract

The structural, mechanical, and electronic properties, as well as stability, elastic anisotropy and effective mass of AlN/GaN/InN in the *Pmn*2_1_ phase were determined using density functional theory (DFT). The phonon dispersion spectra and elastic constants certify the dynamic and mechanical stability at ambient pressure, and the relative enthalpies were lower than those of most proposed III-nitride polymorphs. The mechanical properties reveal that *Pmn*2_1_-AlN and *Pmn*2_1_-GaN possess a high Vickers hardness of 16.3 GPa and 12.8 GPa. *Pmn*2_1_-AlN, *Pmn*2_1_-GaN and *Pmn*2_1_-InN are all direct semiconductor materials within the HSE06 hybrid functional, and their calculated energy band gaps are 5.17 eV, 2.77 eV and 0.47 eV, respectively. The calculated direct energy band gaps and mechanical properties of AlN/GaN/InN in the *Pmn*2_1_ phase reveal that these three polymorphs may possess great potential for industrial applications in the future.

## 1. Introduction

Third-generation semiconductor materials have been of great interest in the past decade because of their importance in scientific research and their industrial applications [1,2,3,4,5,6]. Group III-V compound semiconductors are among the most widely used third-generation semiconductor materials, represented by GaN, AlN, SiC and diamond. These semiconductor materials have some common advantages, such as wide band gap, high electron saturation rate, high radiation resistance, high thermal conductivity, and high electric field [7,8,9,10]. Thus, they have important technological applications in optoelectronic devices, light-emitting diodes (LEDs), high-frequency electronic devices, radiation-resistant electronic devices and high-power electronic devices.

First-principles calculations based on density functional theory (DFT) are among the most reliable and popular microscopic theories in material science. This method has a high ability to predict the material structures and properties [11,12,13,14,15,16,17,18,19,20]. Yang et al. [21] predicted a novel high-pressure superhard BN phase at high pressure through a developed particle swarm optimization (PSO) algorithm. The calculations revealed that its Vickers hardness is 47 GPa, which is characteristic of a superhard material. Liu et al. [22] proposed three new metastable phases (*P*6_4_22, *C*222, *Pbca*, and *I*$\bar{4}$3d) for AlAs. The electronic band structure calculation reveals *I*$\bar{4}$3d-AlAs is a direct semiconductor material with energy band gap of 1.76 eV, whereas *C*222- and *P*6_4_22-AlAs are indirect semiconductor materials with band gaps of 0.47 eV and 1.36 eV, respectively. Xu et al. [23] calculated the mechanical and thermodynamic properties of AlN/AlP/AlAs compounds in wurtzite, zinc-blende, and rock-salt structures through first-principles calculations. They found the hardness and Debye temperatures both decrease with the X (X = N, P, As) atomic number. Zhang et al. [24] studied the physical properties of four III-Nitride compounds, one indirect semiconductor material (*Pm*-3*n*-BN) and three direct semiconductor materials (*Pm*-3*n*-AlN, *Pm*-3*n*-GaN, and *Pm*-3*n*-InN). The band gaps of *Pm*-3*n-*BN/AlN/GaN/InN are 1.04 eV, 2.40 eV, 3.27 eV and 5.87 eV, respectively. Compared with the previous materials (AlGaAs, AlGaN, GaAsP, AlGaInP quaternary semiconductor alloy, or AlGaN and other ternary semiconductor alloys), they save the trouble of making ternary or quaternary semiconductors in semiconductor technology [24]. The materials in the *Pmn*2_1_ phase, such as Li_2_FeSiO_4_, have been recognized as potential cathode materials for Li-ion batteries and have attracted the attention of many researchers [25,26,27] because of their safety, benign element composition and high theoretical energy density. Yang et al. [28] analyzed the structural, elastic, thermodynamic, optical and electronic properties of B_1-*x*_Al*_x_*N in the *Pmn*2_1_ phase by using density functional theory. Chittari [29] studied the dynamic stability of the *Pmn*2_1_ phase of NH_3_BH_3_ by van der Waals-corrected density functional theory.

In this work, we propose two polymorphs of the III-Nitride compound semiconductor in the *Pmn*2_1_ phase, GaN and InN, together with the AlN [30]. Their structural, mechanical, and electronic properties, as well as stability, elastic anisotropy, and effective mass are studied by density functional theory (DFT) [31]. All three polymorphs have direct band gaps and are mechanically and dynamically stable. The two-dimensional (2D) and three-dimensional (3D) perspectives of Young’s modulus, together with universal anisotropic index (*A*^U^) are used to analyze mechanical anisotropy.

## 2. Computational Methods

For the modeling of the AlN/GaN/InN in the *Pmn*2_1_ phase, we use density functional theory (DFT)-based methods [31] realized in the plane-wave pseudopotential approach in the Cambridge Sequential Total Energy Package (CASTEP) codes [32]. The generalized gradient approximation (GGA) of the Perdew-Burke-Ernzerhof (PBE) [33] scheme and the local density approximation (LDA) [34,35] were used to optimize the geometry and calculate elastic constants.

The values of the cutoff energies are set as 280/330/350 eV for AlN/GaN/InN in the *Pmn*2_1_ phase, with k-point samplings with 0.025 Å^−1^ (13 × 3 × 7/11 × 3 × 6/13 × 3 × 7) in the first irreducible Brillouin zone for *Pmn*2_1_-AlN, *Pmn*2_1_-GaN and *Pmn*2_1_-InN. The geometry optimization parameters were determined using the Broyden–Fletcher–Goldfarb–Shenno (BFGS) algorithm [36], with the following convergence tolerance: displacement of atoms during the geometry optimization less than 0.0005 Å, energy change less than 5 × 10^−6^ eV per atom, stress less than 0.02 GPa, and residual force below 0.01 eV/Å. The phonon frequencies were calculated using linear response theory [37]. We used the Heyd–Scuseria–Ernzerhof (HSE06) hybrid functional [38] to calculate electronic band structures and partial density of state (PDOS) based on the optimized geometry.

## 3. Results

### 3.1. Structural Properties

**Figure 1:**
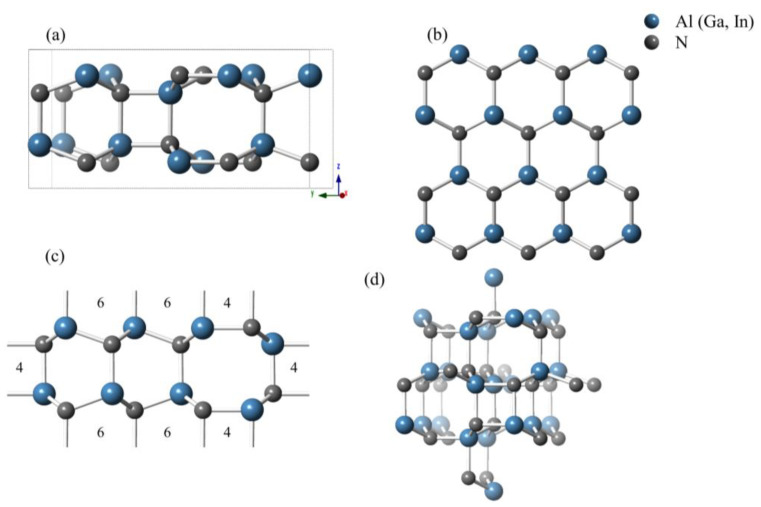
The structures of AlN/GaN/InN in the *Pmn*2_1_ phase at ambient pressure: (**a**) unit cell of the crystal structure; views of the supercell structure along the (**b**) [001] direction, (**c**) [100] direction and (**d**) overview direction.

Figure 1 shows the crystal structure of the predicted *Pmn*2_1_-AlN/GaN/InN in different views and forms. This structure belongs to the *Pmn*2_1_ space group of the orthorhombic system. The structure of *Pmn*2_1_-AlN/GaN/InN consists of sp^3^-bonded rings in three different shapes. Figure 1a,c shows the four-, six-, and eight-membered rings consisting of Al/Ga/In atoms and N atoms along two different views. Figure 1b shows the six-membered rings, which can form a honeycomb-like structure. Figure 1c shows that three four-membered Al/Ga/In-N rings are located by the eight-membered ring, and another four-membered Al/Ga/In-N ring is located by the top of the six-membered ring. There are eight atoms in the conventional cell of *Pmn*2_1_-AlN, and all atoms occupy the crystallographic 2*a* sites in a conventional cell, as follows: Al: (0.0, 0.463, 0.176); (0.5, 0.208, 0.316); (0.5, 0.041, 0.815); (0.5, 0.714, 0.315). N: (0.0, 0.462, 0.798); (0.5, 0.208, 0.692); (0.5, 0.041, 0.193); (0.5, 0.713, 0.695). The atomic Wyckoff positions for *Pmn*2_1_-GaN and *Pmn*2_1_-InN can be obtained by replacing the position of Al with Ga and In atoms.

The optimized equilibrium lattice parameters for AlN/GaN/InN in the *Pmn*2_1_ phase at zero pressure are listed in Table 1. The results show that the calculated values are in great agreement with other theoretical results and experimental results in Table 1, which shows that the present optimization and calculation are reliable. The results obtained by PBE functional are closer to experimental values, so the results obtained by PBE functional are used in this paper. The bond angles of Al-N, Ga-N and In-N in the *Pmn*2_1_ phase range from 87.62 degrees to 117.91 degrees. In *Pmn*2_1_-AlN, *Pmn*2_1_-GaN and *Pmn*2_1_-InN, the bond lengths range from 1.872 Å to 1.935 Å, 1.939 Å to 2.009 Å and 2.170 Å to 2.243 Å, respectively. For zb-AlN (zinc-blende AlN), zb-GaN, and zb-InN, the bond lengths are 1.905 Å, 1.975 Å and 2.205 Å, respectively. For wz-AlN (wurtzite AlN), wz-GaN, and wz-InN, the bond lengths range from 1.901 Å to 1.913 Å, 1.973 Å to 1.981 Å and 2.202 Å to 2.211 Å, respectively. The densities of *Pmn*2_1_-AlN, *Pmn*2_1_-GaN and *Pmn*2_1_-InN are 3.150 g/cm^3^, 5.742 g/cm^3^ and 6.351 g/cm^3^, respectively, which are close to those of AlN/GaN/InN in the wurtzite phase.

### 3.2. Stability and Mechanical Properties

**Figure 2:**
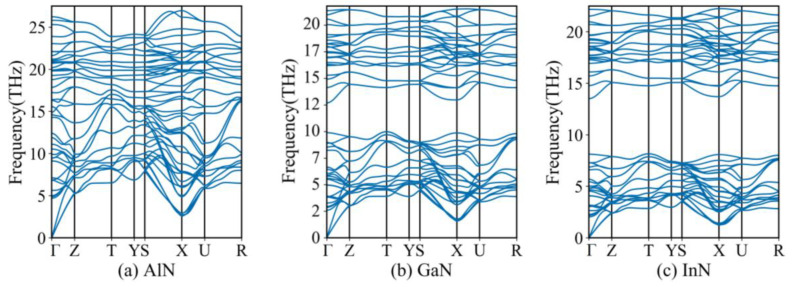
The phonon spectra of (**a**) *Pmn*2_1_-AlN, (**b**) *Pmn*2_1_-GaN and (**c**) *Pmn*2_1_-InN.

The phonon spectra of AlN/GaN/InN in the *Pmn*2_1_ phase were calculated under ambient conditions (see Figure 2) in this work. There is no imaginary frequency throughout the Brillouin zone, which means *Pmn*2_1_-AlN, *Pmn*2_1_-GaN and *Pmn*2_1_-InN are all dynamically stable. The relative enthalpies at zero pressure are also calculated using the following expression:

(1) $$\Delta H=(E_{total}-n_{X}E_{solid}^{X}-n_{N}E_{solid}^{N})/(n_{X}+n_{N})$$

where the $E_{total}$ is the total energy of *Pmn*2_1_-XN (X = Al, Ga, In); the $n_{X}$ is the number of Al/Ga/In atoms in the cell; the $n_{N}$ is the number of N atoms in the cell; $E_{solid}^{X}$is the energy of one X (X = Al, Ga, In) atom in elemental X (X = aluminium, gallium, indium). $E_{solid}^{N}$is the energy of one nitrogen atom in elemental nitrogen. For AlN, the enthalpies relative to zb-AlN and wz-AlN are 0.004 eV per formula unit and 0.045 eV per formula unit. For GaN, the enthalpies relative to zb-GaN and wz-GaN are 0.055 eV per formula unit and 0.066 eV per formula unit. Finally, for InN, the enthalpies relative to zb-InN and wz-InN are 0.066 eV per formula unit and 0.054 eV per formula unit. Compared with other reported III-nitride polymorphs, *Pmn*2_1_-AlN (0.045 eV/f.u.) is more favorable than *Pnma*-AlN (0.232 eV/f.u.) [42], *Cmcm-*AlN (0.206 eV/f.u.) and *Pbca*-AlN (0.075 eV/f.u.) [30]. As seen from the enthalpies relative to wz-GaN, *Pmn*2_1_-GaN (0.066 eV eV/f.u.) is more favorable than *Pnma*-GaN (0.264 eV/f.u.) [43]. The results reveal that *Pmn*2_1_-AlN and *Pmn*2_1_-GaN are more favorable than most proposed polymorphs of AlN and GaN [30,42,43].

Table 2 shows the elastic constants and elastic modulus for AlN/GaN/InN in the *Pmn*2_1_ phase, together with reported calculated and experimental results for comparison [44,45,46]. There are nine independent elastic constants for the orthorhombic phase, namely, *C*_11_*, C*_12_*, C*_13_*, C_22_, C*_23_*, C*_33_*, C*_44_*, C*_55_ and *C*_66_. The mechanical stability criteria [47] of the orthorhombic structure are given as follows:

(2) $$C_{11}>0,C_{12}>0,C_{13}>0,C_{22}>0,C_{23}>0,C_{33}>0,C_{44}>0,C_{55}>0,C_{66}>0,$$

(3) $$\left[C_{11}+C_{22}+C_{33}+2(C_{12}+C_{13}+C_{23})\right]>0,$$

(4) $$\left(C_{11}+C_{22}-2C_{12}\right)>0,\left(C_{11}+C_{33}-2C_{13}\right)>0,\left(C_{22}+C_{33}-2C_{23}\right)>0,$$

The calculated elastic constants of *Pmn*2_1_-AlN, *Pmn*2_1_-GaN, and *Pmn*2_1_-InN indicate that these structures are mechanically stable. According to the elastic constants listed in Table 2, the *C*_11_ values of *Pmn*2_1_-AlN, *Pmn*2_1_-GaN, and *Pmn*2_1_-InN are larger than those of wz-AlN/GaN/InN and zb-AlN/GaN/InN, which means that all three proposed polymorphs possess a better compression resistance in the *x* direction than its corresponding III-nitride in the wurtzite phase and zinc-blende phase. Additionally, the *C*_33_ values of *Pmn*2_1_-AlN/GaN/InN are smaller than those of wz-AlN/GaN/InN and zb-AlN/GaN/InN, which reveal a better deformability along the *z* direction.

The bulk modulus (*B*) and shear modulus (*G*) were calculated by using the Voigt-Reuss-Hill approximation [48], which are defined as *B* = (*B_V_* + *B_R_*)*/*2, *G =* (*G_V_ + G_R_*)*/*2. Where *B_V_* means the Voigt approximation of bulk modulus *B*; *B_R_* is the Reuss approximation of bulk modulus *B*; *G_V_* means the Voigt approximation of shear modulus *G*; and *G_R_* is the Reuss approximation of shear modulus *G*.

The Young’s modulus E is used to offer a measure of the stiffness of a solid. The larger the value of E is, the stiffer the material is [49]. The Young’s modulus *E* and Poisson’s ratio *v* were determined as follows [50]: *E =* 9*BG*/(3*B + G)*, *v =* (*3B − 2G*)*/*[*2(3B + G*)]. The obtained results are listed in Table 2. The calculated bulk modulus *B* and shear modulus *G* of *Pmn*2_1_-AlN/GaN/InN are slightly less than those of wz-AlN/GaN/InN and zb-AlN/GaN/InN. The shear modulus is less than the bulk modulus for *Pmn*2_1_-AlN/GaN/InN. The values of *B/G* and Poisson’s ratio are associated with brittleness or ductility. If *B/G* > 1.75 [51], a material is characterized as ductile; otherwise, the material has a brittle character. Poisson’s ratio <0.26 indicates brittle compounds [52]. Obviously, *Pmn*2_1_-AlN and *Pmn*2_1_-GaN exhibit brittle character, whereas *Pmn*2_1_-InN behaves in a ductile manner.

The Vickers hardness (Hv) was calculated by adopting Chen’s formula based on an empirical scheme [53]:

(5) $$H_{v}=2{\left(k^{2}G\right)}^{0.585}-3;\;k=G/B$$

The calculated and experimental hardness values are presented in Table 2. The calculated hardness reveals that the proposed *Pmn*2_1_-AlN and *Pmn*2_1_-GaN possess a high Vickers hardness of 16.3 GPa and 12.8 GPa; however, *Pmn*2_1_-InN possesses a Vickers hardness of 3.9 GPa. The results show that in the *Pmn*2_1_, zinc-blende and wurtzite phases, AlN possesses the highest hardness among these three polymorphs, the hardness of GaN is slightly lower than that of AlN, and InN possesses the lowest hardness.

### 3.3. Mechanical Anisotropic Properties

**Figure 3:**
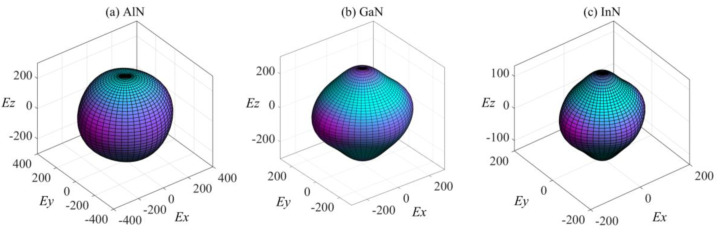
The surface construction of Young’s modulus for (**a**) *Pmn*2_1_-AlN, (**b**) *Pmn*2_1_-GaN and (**c**) *Pmn*2_1_-InN.

It is well-known that the anisotropy of elasticity is an important implication in engineering science and crystal physics. Figure 3 shows the variation in Young’s modulus for *Pmn*2_1_-AlN/GaN/InN with three-dimensional crystallographic directions. The directional dependence of the Young’s modulus *E* for the orthorhombic crystal is [54]:

(6) $$\begin{array}{c} E^{-1}=m_{1}^{4}S_{11}+m_{2}^{4}S_{22}+m_{3}^{4}S_{33}+2m_{1}^{2}m_{2}^{2}S_{12}+2m_{1}^{2}m_{3}^{2}S_{13}+\\ 2m_{2}^{2}m_{3}^{2}S_{23}+m_{1}^{2}m_{2}^{2}S_{66}+m_{1}^{2}m_{3}^{2}S_{55}+m_{2}^{2}m_{2}^{2}S_{44} \end{array}$$

where *S*_ij_ refers to the elastic compliance constants and *m*_1_, *m*_2_, and *m*_3_ are the direct cosines of the [*u v w*] direction. Figure 3 reveals that *Pmn*2_1_-AlN possesses the smallest elastic anisotropy and largest Young’s modulus among these three polymorphs. *Pmn*2_1_-GaN and *Pmn*2_1_-InN show a larger elastic anisotropy in the *yz* plane and *xz* plane. The *E*_max_*/E*_min_ ratios of *Pmn*2_1_-AlN, *Pmn*2_1_-GaN and *Pmn*2_1_-InN are 1.26, 1.26 and 1.32, respectively. The calculated results show that the elastic anisotropy of the Young’s modulus for *Pmn*2_1_-AlN/GaN/InN reflects a slight increase, from *Pmn*2_1_-AlN to *Pmn*2_1_-GaN and then to *Pmn*2_1_-InN.

To further understand the elastic anisotropy of the Young’s modulus along different directions, the dependence of the Young’s modulus on orientation was investigated by taking the tensile axis within a specific plane. Let *α* be the angle between [1 0 0] and [*u v* 0] for the *xy* plane; then, the Young’s modulus between [1 0 0] and [*u v* 0] for the *xy* plane can be expressed as:

(7) $$E^{-1}=S_{11}\cos^{4}\alpha +S_{22}\sin^{4}\alpha +2S_{12}\sin^{2}\alpha \cos^{2}\alpha +S_{66}\sin^{2}\alpha \cos^{2}\alpha$$

Let *β* be the angle between [0 0 1] and [*u* 0 *w*] for the (0 1 0) plane; then, the Young’s modulus between [0 0 1] and [*u* 0 *w*] for the *xz* plane can be expressed as:

(8) $$E^{-1}=S_{11}\sin^{4}\beta +S_{33}\cos^{4}\beta +[2S_{13}\sin^{2}2\beta +S_{55}\sin^{2}2\beta ]/4$$

Let *γ* be the angle between [0 0 1] and [0 *v w*] for the (0 0 1) plane; Then, the Young’s modulus between [0 0 1] and [0 *v w*] for the *xy* plane can be expressed as:

(9) $$E^{-1}=S_{22}\sin^{4}\gamma +S_{33}\cos^{4}\gamma +\left[2S_{33}\sin^{2}2\gamma +S_{44}\sin^{2}2\gamma \right]/4$$

**Figure 4:**
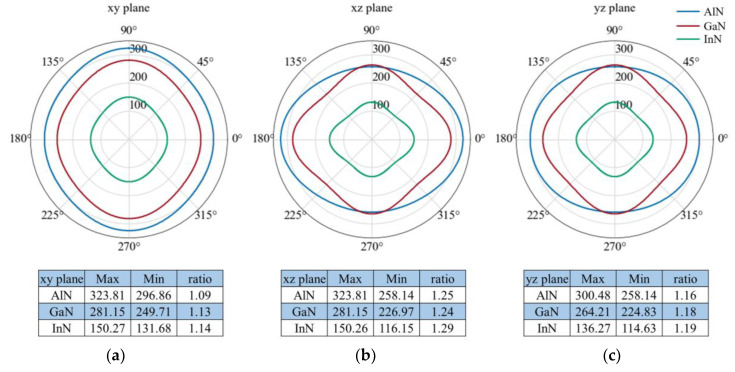
Two-dimensional representation of the Young’s modulus in the *xy* plane, *xz* plane and *yz* plane for (**a**) *Pmn*2_1_-AlN, (**b**) *Pmn*2_1_-GaN and (**c**) *Pmn*2_1_-InN.

Two-dimensional representations of the Young’s modulus for *Pmn*2_1_-AlN/GaN/InN are illustrated in Figure 4. The lines representing *Pmn*2_1_-AlN, *Pmn*2_1_-GaN, and *Pmn*2_1_-InN are shown in blue, red, and green, respectively. From Figure 3 and Figure 4, we find that *Pmn*2_1_-InN exhibits the smallest elastic anisotropy in the Young’s modulus and that *Pmn*2_1_-AlN exhibits the largest elastic anisotropy. For these three primary planes, the maximum values for *Pmn*2_1_-AlN, *Pmn*2_1_-GaN, and *Pmn*2_1_-InN all occur in the *xz* plane and *xy* plane, and the minimum values occur in the *yz* plane. In addition, the *xy* plane of *Pmn*2_1_-AlN, *Pmn*2_1_-GaN, and *Pmn*2_1_-InN exhibits the smallest elastic anisotropy in the Young’s modulus, and the *E*_max_*/E*_min_ ratios are 1.09, 1.13 and 1.14, respectively. The *xz* plane exhibits the greatest elastic anisotropy in the Young’s modulus for *Pmn*2_1_-AlN/GaN/InN.

In addition, apart from the surface construction and two-dimensional representation of Young’s modulus, the universal anisotropic index *A*^U^ [55] is also calculated for deeper investigation in this work. *A*^U^ is defined as *A*^U^ = 5*G_V_/G_R_ + B_V_/B_R_ −* 6, where *B*_V_ (*B*_R_) and *G*_V_ (*G*_R_) represent the symbols of the bulk modulus and shear modulus at Voigt (Reuss) bounds, respectively. The *A*^U^ of *Pmn*2_1_-AlN/GaN/InN are 0.0454, 0.0801 and 0.1006, respectively. The calculated *A*^U^ is similar to the three-dimensional and two-dimensional representation of the Young’s modulus, it also shows an increasing tendency with the group III element (Al, Ga, In) atomic number.

### 3.4. Electrical and Thermal Properties

**Figure 5:**
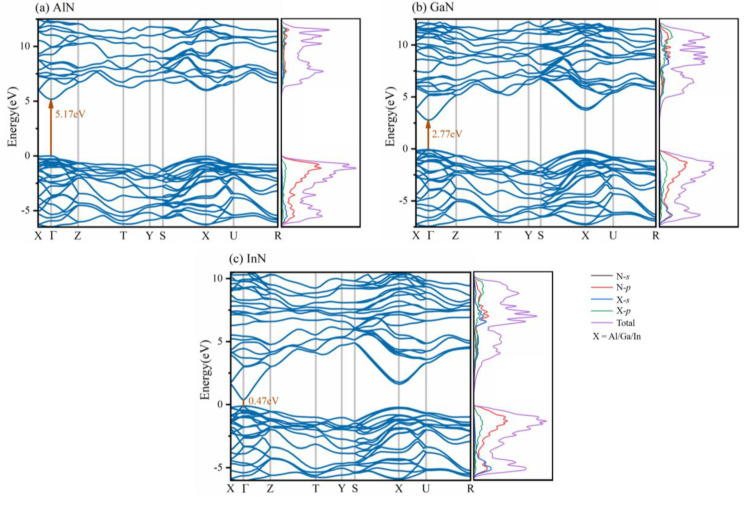
Electronic band structure and Partial density of states for (**a**) *Pmn*2_1_-AlN, (**b**) *Pmn*2_1_-GaN and (**c**) *Pmn*2_1_-InN.

The energy band structure of the material determines a variety of properties, especially its electronic and optical properties. The electronic band structure, together with partial density of state (PDOS) of *Pmn*2_1_-AlN/GaN/InN are shown in Figure 5. All three proposed compounds are semiconductor materials with direct bandgaps at G points of 5.17 eV (*Pmn*2_1_-AlN), 2.77 eV (*Pmn*2_1_-GaN) and 0.47 eV (*Pmn*2_1_-InN), notably *Pmn*2_1_-AlN and *Pmn*2_1_-GaN, which are wide bandgap semiconductors [56]. In a light emitting diode, only the direct transition process can produce light, which is the main transition method for direct semiconductors. The wavelength of light is mostly determined by the energy band gap of the semiconductors [57]. The band gap of *Pmn*2_1_-GaN is 2.77 eV, which is lower than that of wz-GaN (3.4 eV) [58] and corresponds to the blue light region, making it a potential material for blue LEDs without adulteration. The energy band gaps of *Pmn*2_1_-AlN and *Pmn*2_1_-InN correspond to the ultraviolet region and infrared region, respectively. This suggests that *Pmn*2_1_-AlN and *Pmn*2_1_-InN have the potential to produce optoelectronic devices.

The lines represent the total DOS, N-*s*, N-*p*, X-*s*, and X-*p* (X = Al, Ga, In) are set to purple, black, red, blue and green, respectively. According to the PDOS diagram of *Pmn*2_1_-AlN/GaN/InN, the density of states mainly comes from N-*p* orbitals. Below 0 eV, the total DOS in the valence band originates mainly from N-*p* orbitals for these three compounds. Above 0 eV, the N-*p*, X-*s* and X-*p* orbitals (X = Al, Ga, In) contribute greatly and overlap with each other. In addition, the N-*s* orbitals contribute the smallest proportion in the valance band and conduction band. For *Pmn*2_1_-AlN/GaN/InN, the peaks are all present in the energy region close to 0 eV (−2 to 0 eV), and the DOS is mainly due to the contributions from N-*p* orbitals; the contribution of other electron orbitals is relatively small. These DOS peaks depend on the N-*p*/X-*p* (X = Al, Ga, In) bonding orbital contribution. The results show that covalent N-X (X = Al, Ga, In) interactions exist.

The effective mass is also calculated by quadratic polynomial fitting of valence and conduction bands along the *x*, *y*, and *z* directions. The effective mass can be determined as follows: ${{(m}^{*})}^{-1}=(1/{ћ}^{2})(\partial^{2}E/\partial k^{2})$. The calculated hole effective mass and electron effective mass of *Pmn*2_1_-AlN/GaN/InN, zb-AlN/GaN/InN and the experimental values for comparison are listed in Table 3. The electron effective mass of these three proposed III-nitride polymorphs along the *x*, *y* and *z* directions gradually decrease, whereas the electron effective mass along these three directions are almost the same. For *Pmn*2_1_-AlN/GaN/InN, the largest hole effective mass occurs along the *y* direction, and the smallest occurs along the *z* direction. For *Pmn*2_1_-AlN, the hole effective mass and the electron effective mass along the *x*, *y* and *z* directions are larger than those of *Pmn*2_1_-GaN, *Pmn*2_1_-InN and zb-AlN/GaN/InN. For *Pmn*2_1_-GaN, the hole effective mass along the *y* direction is close to that of *Pmn*2_1_-InN. Finally, for *Pmn*2_1_-InN, the hole effective mass along the *z* direction is much smaller than that of zb-InN, and the electron effective mass of *Pmn*2_1_-InN along all directions is close to that of zb-InN.

## 4. Conclusions

In summary, we investigated the structural, stability, mechanical and electronic properties of *Pmn*2_1_-AlN/GaN/InN. *Pmn*2_1_-AlN/GaN/InN are mechanically and dynamically stable. The relative enthalpies of *Pnma*-AlN and *Pmn*2_1_-GaN are more favorable than those of most predicted III-nitride polymorphs. The elastic constants indicate that *Pmn*2_1_-AlN/GaN/InN possess better deformation resistance properties in the *x* direction and better deformability along the *z* direction than wz-AlN/GaN/InN and zb-AlN/GaN/InN. The calculated *H_v_* values of *Pmn*2_1_-AlN and *Pmn*2_1_-GaN reveal that *Pmn*2_1_-AlN and *Pmn*2_1_-GaN possess a high hardness of 16.3 GPa and 12.8 GPa, respectively. *Pmn*2_1_-AlN, *Pmn*2_1_-GaN and *Pmn*2_1_-InN exhibit similar elastic anisotropies. The electron effective mass of *Pmn*2_1_-InN is smaller along all three directions than that of zb-InN. In addition, the hole effective mass of *Pmn*2_1_-GaN and *Pmn*2_1_-InN along the *z* direction are much smaller than those of zb-GaN and zb-InN. *Pmn*2_1_-AlN/GaN/InN are direct semiconductor materials with energy band gaps of 5.17 eV (*Pmn*2_1_-AlN), 2.77 eV (*Pmn*2_1_-GaN) and 0.47 eV (*Pmn*2_1_-InN). Ultimately, *Pmn*2_1_-AlN, *Pmn*2_1_-GaN and *Pmn*2_1_-InN may have great potential industrial applications in the future due to their superior electronic and mechanical properties.

## Figures and Tables

**Table 1:** Lattice constants *a*, *b*, and *c* (Å) and density *ρ* (g cm^−3^) for AlN/GaN/InN in the *Pmn*2_1_ phase.

Structure		*a*	*b*	*c*	*ρ*
*Pmn*2_1_-AlN	GGA	3.130	10.832	5.099	3.150
*Pmn*2_1_-GaN	GGA	3.231	11.202	5.352	5.742
*Pmn*2_1_-InN	GGA	3.617	12.528	5.946	6.351
wz-AlN	GGA	3.125		5.009	3.206
	LDA	3.066		4.905	3.408
	Exp. ^a^	3.111		4.978	
wz-GaN	GGA	3.227		5.258	5.865
	LDA	3.158		5.149	6.254
	Exp. ^a^	3.192		5.166	
wz-InN	GGA	3.611		5.842	6.485
	LDA	3.531		5.714	6.936
	Exp. ^a^	3.533		5.639	
zb-AlN	GGA	4.399			3.198
	LDA	4.309			3.404
	Exp. ^b^	4.370			
zb-GaN	GGA	4.561			5.862
	LDA	4.466			6.248
	Exp. ^c^	4.490			
zb-InN	GGA	5.092			6.482
	LDA	4.977			9.642

^a^ Reference [39]; ^b^ Reference [40]; ^c^ Reference [41].

**Table 2:** The calculated elastic constants (GPa) and elastic modulus (GPa) of *Pmn*2_1_-AlN/GaN/InN.

Structure	*C*_11_	*C*_12_	*C*_13_	*C*_22_	*C*_23_	*C*_33_	*C*_44_	*C*_55_	*C*_66_	*B*	*G*	*B/G*	*E*	*ν*	*H_v_*
*Pmn*2_1_-AlN	383	122	97	352	89	294	117	112	115	181	116	1.56	288	0.235	16.3
wz-AlN	376	118	88			353	113		129	188	125	1.50	307	0.227	18.0
	345 ^a^	125	120			395	118		110						18 ^d^
zb-AlN	276	143					181			187	121	1.54	299	0.233	17.0
	304 ^b^	160					193								
*Pmn*2_1_-GaN	333	109	84	299	75	294	87	85	94	162	97	1.68	241	0.251	12.8
wz-GaN	322	102	69			361	89		110	165	107	1.54	264	0.233	15.5
	390 ^c^	145	106			398	105		123						15.1 ^d^
zb-GaN	249	127					151			168	105	1.60	260	0.242	14.5
	293 ^b^	159					155								
*Pmn*2_1_-InN	207	91	74	185	68	168	42	42	47	113	48	2.38	125	0.316	3.9
wz-InN	194	90	64			202	48		52	114	54	2.12	140	0.296	5.6
	223 ^b^	115	92			224	48								5.1 ^d^
zb-InN	154	101					87			119	54	2.18	142	0.301	5.3
	187 ^b^	125					86								

^a^ Reference [39]; ^b^ Reference [44]; ^c^ Reference [46]; ^d^ Reference [53].

**Table 3:** The calculated effective mass (in m_0_) along the *x*, *y*, and *z* directions for *Pmn*2_1_-AlN, *Pmn*2_1_-GaN, and *Pmn*2_1_-InN.

***x*** **Direction**	**Hole Effective Mass**	**Electron Effective Mass**
	*Pmn*2_1_/zinc-blende/Ref	*Pmn*2_1_/zinc-blende/Ref
AlN	3.29/1.23/1.02 ^a^	0.32/0.33/0.23 ^a^
GaN	1.95/0.90/0.80 ^a^	0.16/0.14/0.14 ^a^
InN	1.14/0.94/1.08 ^a^	0.08/0.08/0.13 ^a^
***y*** **Direction**	**Hole Effective Mass**	**Electron Effective Mass**
	*Pmn*2_1_/zinc-blende	*Pmn*2_1_/zinc-blende/Ref
AlN	5.31/1.23	0.31/0.33/0.23 ^a^
GaN	2.58/0.90	0.16/0.14/0.14 ^a^
InN	2.55/0.94	0.08/0.08/0.13 ^a^
***z*** **Direction**	**Hole Effective Mass**	**Electron Effective Mass**
	*Pmn*2_1_/zinc-blende/Ref	*Pmn*2_1_/zinc-blende/Ref
AlN	0.27/1.23/1.33 ^b^	0.30/0.33/0.23 ^a^
GaN	0.15/0.90/0.81 ^b^	0.15/0.14/0.14 ^a^
InN	0.09/0.94/0.84 ^b^	0.08/0.08/0.13 ^a^

^a^ Reference [59]; ^b^ Reference [60].
